# Respiratory Variations in Electrocardiographic R-Wave Amplitude during Acute Hypovolemia Induced by Inferior Vena Cava Clamping in Patients Undergoing Liver Transplantation

**DOI:** 10.3390/jcm8050717

**Published:** 2019-05-20

**Authors:** Hee-Sun Park, Sung-Hoon Kim, Yong-Seok Park, Robert H. Thiele, Won-Jung Shin, Gyu-Sam Hwang

**Affiliations:** 1 Department of Anesthesiology and Pain Medicine, University of Ulsan College of Medicine, Asan Medical Center, 05505 Seoul, Korea; heespark@amc.seoul.kr (H.-S.P.); parkys@amc.seoul.kr (Y.-S.P.); wjshin@amc.seoul.kr (W.-J.S.); kshwang@amc.seoul.kr (G.-S.H.); 2 Departments of Anesthesiology and Biomedical Engineering, University of Virginia School of Medicine, Charlottesville, VA 22903, USA; rht7w@virginia.edu

**Keywords:** Brody effect, electrocardiographic variation, R-wave amplitude, hemodynamic monitoring, pulse pressure variation

## Abstract

The aim of this study was to analyze whether the respiratory variation in electrocardiogram (ECG) standard lead II R-wave amplitude (ΔRDII) could be used to assess intravascular volume status following inferior vena cava (IVC) clamping. This clamping causes an acute decrease in cardiac output during liver transplantation (LT). We retrospectively compared ΔRDII and related variables before and after IVC clamping in 34 recipients. Receiver operating characteristic (ROC) curve and area under the curve (AUC) analyses were used to derive a cutoff value of ΔRDII for predicting pulse pressure variation (PPV). After IVC clamping, cardiac output significantly decreased while ΔRDII significantly increased (*p* = 0.002). The cutoff value of ΔRDII for predicting a PPV >13% was 16.9% (AUC: 0.685) with a sensitivity of 57.9% and specificity of 77.6% (95% confidence interval 0.561 – 0.793, *p* = 0.015). Frequency analysis of ECG also significantly increased in the respiratory frequency band (*p* = 0.016). Although significant changes in ΔRDII during vena cava clamping were found at norepinephrine doses <0.1 µg/kg/min (*p* = 0.032), such changes were not significant at norepinephrine doses >0.1 µg/kg/min (*p* = 0.093). ΔRDII could be a noninvasive dynamic parameter in LT recipients presenting with hemodynamic fluctuation. Based on our data, we recommended cautious interpretation of ΔRDII may be requisite according to vasopressor administration status.

## 1. Introduction

In patients under mechanical ventilation, positive-pressure inspiration induces cyclic changes in vena-cava, pulmonary arterial and aortic blood flow. As a result, right ventricular stroke volume decreases during the inspiratory phase, leading to a decrease in left ventricular output after only two or three beats [1]. These heart–lung interactions and cyclic changes influence arterial pulse pressure (PPV) [2] and stroke volume (SVV) [3], both of which can be used to assess fluid responsiveness over three or more breaths [4]. To acquire these dynamic indices, invasive arterial catheterization and specific devices are necessary. They may not always be available, however, due to cost and device limitations. Instead, noninvasive hemodynamic monitoring methods have been developed such as plethysmography [5] or bioreactance technology [6].

The electrocardiogram (ECG), a noninvasive monitoring, is routinely used in the most operating rooms and intensive care units (ICUs) [7]. Since the Brody effect was presented, wherein the QRS amplitude changes according to intraventricular volume, [8], much research has found that morphological changes in the ECG may reflect cardiac preload [9,10,11] due to electrical inhomogeneity remote from the heart and its effect on the transmission of myocardium depolarization to the body surface. Cannesson et al. [12] has successfully reported that respiratory variation in ECG lead II R-wave amplitude (ΔRDII) is closely related to PPV (*r* = 0.79). These researchers focused on respiratory variation in ECG signals during mechanical ventilation as a dynamic preload index in a critical care setting. Previous studies on the ΔRDII in humans have been performed under relatively static circumstances such as an early stage of cardiac surgery [13] or post-operative ICU [12]. However, no report has yet assessed ΔRDII under severe hemodynamic fluctuation during acute central hypovolemia or vasopressor use. In liver transplantation (LT) surgery, inferior vena cava (IVC) clamping causes a sudden decrease in venous return and cardiac output (CO) that results in patients shifting to preload-dependent status [14,15]. We thereby examined the usefulness of ΔRDII as a surrogate index of volume status in surgical patients, as well as whether ΔRDII was associated with PPV or SVV. We also assessed its performance in relation to the use of vasopressors. 

## 2. Methods

### 2.1. Study Population and Data Analysis

This study involved 37 patients who underwent elective, living-donor liver transplantation surgery at Asan Medical Center between June 2016 and January 2017. After the institutional review board’s approval (IRB No. 2018-0194), patient permissions were waived because of the retrospective nature of the current study. This manuscript adheres to applicable STROBE guidelines. We excluded patients with an arrhythmia that prevented measurement of an exact R amplitude such as atrial fibrillation, atrial flutter, or left bundle branch block. Emergency and/or dual-donor LT operations were excluded. Cases with incomplete recording of hemodynamic data were also excluded. The vital data with noisy ECG such as baseline wandering was excluded because of difficulty in measurement. In all cases, general anesthesia was performed according to the standardized protocol of our institution [16,17,18,19], as follow; anesthesia was induced with thiopental, midazolam, fentanyl and rocuronium and was maintained with the use of 4–5 vol% desflurane, 50% oxygen/air, and continuous infusion with fentanyl and rocuronium. Mechanical ventilation was performed without positive end-expiratory pressure, using a constant tidal volume of 6–8 mL/kg and a constant end-tidal carbon dioxide tension of 30–35 mmHg. If necessary, continuous norepinephrine was started (initial dose 0.05–0.1 µg/kg/min) to maintain mean arterial pressure (MAP) above the 65 mmHg mark. 

Five-lead electrocardiography was used and invasive radial arterial pressure was also measured. The arterial line was connected to the Vigileo-FloTrac^TM^ system (Edwards Lifescience, Irvine, CA, US) and the transducer was leveled at the mid-axillary line. Through arterial pulse wave analysis, we obtained CO_FT_, stroke volume (SV_FT_) and stroke volume variation (SVV). A 7.5 Fr pulmonary arterial catheter (PAC) (Swan-Ganz CCOmbo CCO/SvO2/CEDV, Edwards Lifesciences, Irvine, CA, US), was inserted via a 9 Fr introducer sheath into the internal jugular vein and advanced to a wedged position under the guidance of a pressure curve. The pulmonary artery catheter was connected to a Vigilance monitor (Edwards Lifesciences, Irvine, CA, US) in stat mode with measurements updated every 54 s. This mode provided continuous cardiac output (CO_SG_), as well as stroke volume (SV_SG_). Central venous pressure (CVP) and pulmonary capillary wedge pressure (PCWP) were also recorded. 

**Figure 1:**
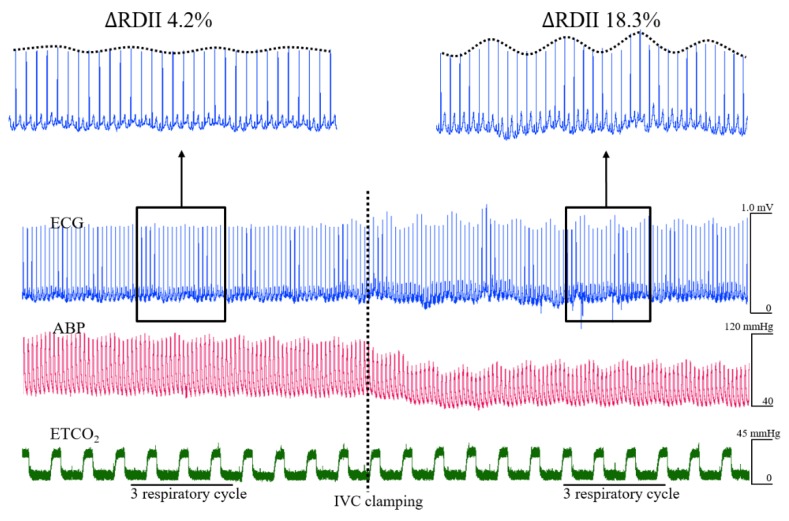
Representative plot of beat-to-beat changes to electrocardiogram (ECG) and arterial blood pressure (ABP) during inferior vena cava (IVC) clamping procedure. Accompanying an acute decrease in ABP, respiratory variation in ECG R-wave amplitude (∆RDII) was markedly augmented. To calculate respiratory variations in RDII, three consecutive respiratory cycles before and after IVC clamping were selected.

All hemodynamic data were continuously recorded at a 1000-Hertz sampling rate, beginning before the induction of anesthesia and continuing until the end of surgery. Intraoperative hemodynamic data were collected via Windaq software (DATAQ Instruments, Akron, OH, US), and stored in an operating room database [20]. For the current analysis, approximately 20 minutes of data before and after the IVC clamping were extracted (Figure 1). Our institute annotated IVC clamping time during surgery, and selected data were reviewed by visual inspection. Windaq files were then transferred to data analysis software LabChart^®^8 Pro (version8.1.9, ADInstruments, Colorado Springs, CO, US) to measure beat-to-beat ECG RDII. A corresponding period of radial arterial pressure was chosen for analysis and calculation of PPV [2]. Standard RDII values were recorded, analyzed offline, and calculated as follows [12]:

(1) $$\Delta \mathrm{RDII}\;\left(\%\right)\;=\;100\times \;\;\frac{maximum\;RDII-minimum\;RDII\;}{\left(\frac{maximum\;RDII+minimum\;RDII}{2}\right)\;}$$

All measurements and calculations were repeated for three consecutive respiratory cycles and averaged for statistical analysis. 

In addition, we conducted an ECG frequency domain analysis at respiraotry (0.13–0.2 Hz), and cardiac frequency (0.75–2.5 Hz). The Fast Fourier Transform (FFT) analysis interval was 30 seconds from the starting point of ΔRDII calculation. We adjusted the spectrum view setting in the spectrum view window of LabChart^®^8 Pro [21]. The data was displayed as amplitude density, and area under the curve (AUC) values of FFT was then calculated for comparison using open-source software ImageJ. 

### 2.2. Statistical Analysis

Demographics and hemodynamic data are expressed as means with standard deviation or number (percentage). A preliminary study of ΔRDII (*n* = 8) was conducted to determine the necessary sample size was to detect a projected difference of 3% in ΔRDII before and after IVC clamping with an SD of 5% (type I error of 0.05 power of 0.9). It was calculated that 32 patients would be required. We aimed to enroll 37 patients to account for possible attrition.

Paired *t*-tests or Wilcoxon signed-rank tests were used for comparisons between hemodynamic values before and after IVC clamping, and between groups at each phase. Receiver operating characteristic (ROC) curves were constructed to evaluate the ΔRDII and other variables to predict whether patient’ PPV was greater than 13% or less than or equal to 13%, according to previous reports [12,22]. AUC values of ROC were calculated, and we performed a Bland–Altman analysis [23] to assess the agreement between ΔRDII and PPV. The mean difference between the two methods (bias) was estimated as the mean of all individual differences. The 95% limits of agreement are estimated as a difference from the mean (± 2 SD). All data analyses were performed with the use of SPSS version 22 (SPSS Inc, Chicago, IL, US), and MedCalc version 13.1.1 (MedCalc Software, Mariakerke, Belgium). A *P* value of less than 0.05 was considered statistically significant.

## 3. Results

We evaluated 37 patients who underwent elective liver transplantation with full recordings of vital data during the surgery. Three patients were excluded due to signal noise or incomplete vital data, leaving us with 34 patients. Baseline demographic data and intraoperative norepinephrine use are listed in Table 1. Changes in hemodynamic variables and ΔRDII before and after IVC clamping are listed in Table 2. ΔRDII, SVV, and PPV were significantly increased after IVC clamping (Table 2). Cardiac output and stroke volume derived from arterial pressure waveform analysis and Swan-Ganz catheter (CO_FT,_ SV_FT_, CO_SG_, and SV_SG_) were significantly decreased after IVC clamping.

**Figure 2:**
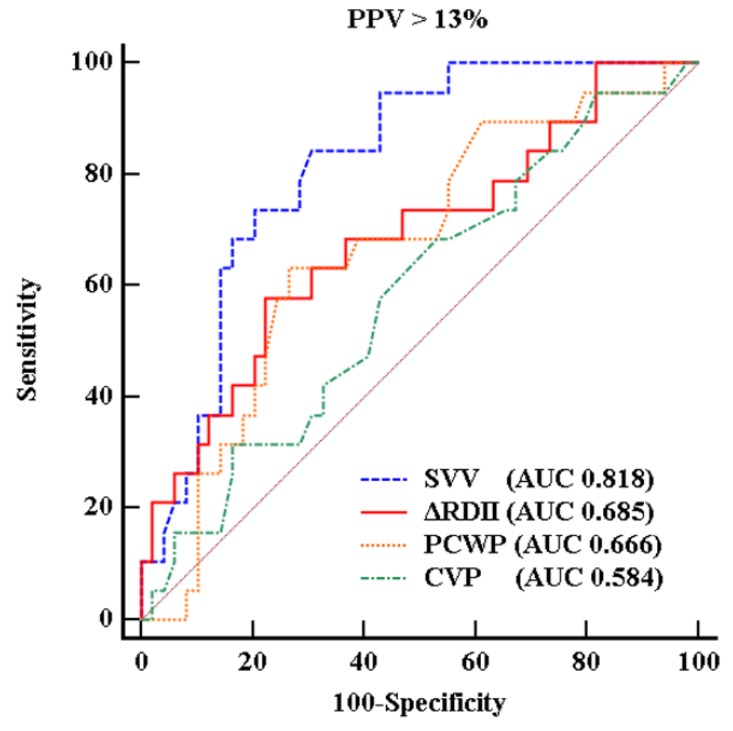
Receiver operating characteristic (ROC) curves. Ability of variation of R-wave amplitude in lead II (ΔRDII) to predict a pulse pressure variation (PPV) > 13% (a sensitivity, 57.9% and a specificity, 77.6%). Stroke volume variation (SVV) showed a sensitivity of 81.3% and a specificity of 83.3%. Pulmonary capillary wedge pressure (PCWP) and central venous pressure (CVP) had a sensitivity of 31.3%, 68.4% and a specificity of 83.3% and 55.6%.

**Figure 3:**
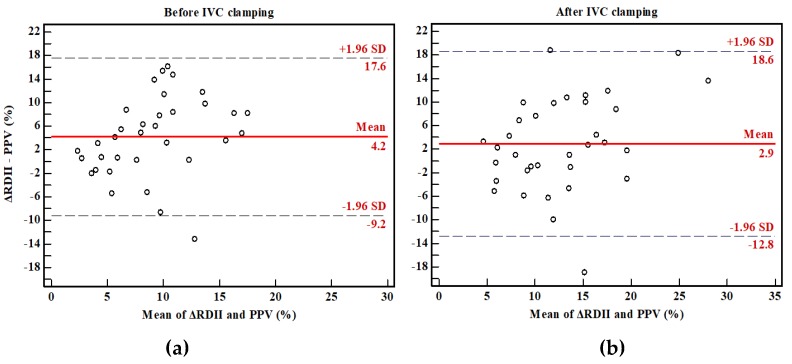
Bland–Altman analyses comparing variation of R-wave amplitude in lead II (ΔRDII) and pulse pressure variation (PPV) during before (**a**) and after (**b**) IVC clamping. The dotted lines represent the average difference between ΔRDII and PPV (i.e., bias) and the solid lines represent the upper and lower 95% confidence limits of agreement.

Results from the ROC curve for the prediction of PPV greater than 13% (Figure 2) demonstrated that the cutoff value therein for ΔRDII was 16.9%, with a sensitivity of 57.9% and a specificity of 77.6% (AUC = 0.685, standard error (SE) = 0.076, 95% confidence interval (CI) 0.561–0.793, *p* = 0.015). The optimal threshold value of SVV was 9.1 % with a sensitivity of 81.3% and a specificity of 83.3% (AUC 0.818, 95% CI 0.706–0.901, *p* < 0.001). While the AUCs of CVP and PCWP were 0.584 with a sensitivity 31.3% and a specificity of 83.3% and 0.666 with a sensitivity of 68.7% and a specificity of 55.6% (95% CI 0.458–0.702 and 0.542–0.776, *p* = 0.267 and 0.017, respectively). Bland–Altman analysis showed that agreement between ΔRDII and PPV was 4.2% with a SD of 6.8% for before IVC clamping, and 2.9% with a SD of 8.0% for after IVC clamping (Figure 3).

At the time of IVC clamping, most patients (31/34) received continuous norepinephrine infusion. The patients were divided into two groups according to whether norepinephrine infusion dose was at least 0.1 µg/kg/min or not (Table 3). ΔRDII, SVV, PPV, and PCWP changed significantly when the norepinephrine dose less than 0.1 µg/kg/min or none. However, changes associated with a norepinephrine dose of more than 0.1 µg/kg/min were not statistically significant (*p* = 0.093).

Frequency analysis of ECG were computed for 30 s before and after IVC clamping. There was a significant increase in respiratory frequency after IVC clamping (*p* = 0.016), which was consistent with the results of ΔRDII and regardless of norepinephrine use (Table 2).

## 4. Discussion

The major finding of this study was that ΔRDII could reflect the decrease in cardiac output after IVC clamping in patients undergoing LT who were administered norepinephrine less than 0.1 µg/kg/min or none. ΔRDII was also related to PPV; the cutoff value of ΔRDII was 16.9% for predicting a PPV above 13%. If ΔRDII monitoring can be performed in a noninvasive manner in real time, it can be an attractive method for roughly assessing volume statues of patients without requiring invasive monitoring.

The concept of the ΔRDII as a dynamic parameter is theoretically based on the Brody effect [8] and the cardio-pulmonary interaction with the ECG R-wave amplitude induced by positive pressure ventilation, as suggested by Cannesson et al. [12]. In this study, cardiac output and stroke volume significantly decreased, while ΔRDII, SVV, and PPV significantly increased for central hypovolemia status after IVC clamping. In contrast to dynamic indices, CVP, a static parameter, did not change significantly. The magnitude of changes in SVV and PPV were greater than that of ΔRDII, at 59%, 64% and 29%, respectively. These findings were consistent with a previous study [24], comparing PPV and ΔRDII in pigs during hypovolemia and normovolemia. These researchers theorized that differences in the magnitude of changes were due to arterial elastance and catecholamine effects on PPV and SVV during hypovolemia [24,25]. The fact that a higher baseline ΔRDII than PPV and a different magnitude of change to ΔRDII in this study might contribute to the results of the ROC analysis, which could explain why our ΔRDII threshold was higher than of previous reports [12].

Previous studies reporting absolute RDII changes could not predict the increase of blood volume (about 300 mL) by passive leg raise [22]. There were also conflicting data in reports regarding an inverse relationship between central volume and R-wave or QRS amplitude [26,27]. ΔRDII or respiratory change of R-wave amplitude was not investigated in these studies. Current and previous studies have found that ΔRDII is correlated with certain dynamic indices such as SVV measured by trans-esophageal echocardiography or PPV. These values could be useful in preload-dependent patients, although not with preload-independent patient [9,12,13,24]. The difference between IVC clamping periods in our Bland–Altman analysis may imply that ΔRDII can be both of a higher sensitivity and lower specificity for hypovolemia. Because a preload-independent heart will not experience large respiratory variations in cardiac volume, large variation in R wave amplitude will not be observed in these patients [12,13,28].

One confounding factor of the current study may be the norepinephrine infusions. Patients undergoing LT are known to have hyper-dynamic circulation with low systemic vascular resistance, usually accompanied by large volumes of blood loss and intravascular fluid shift. These characteristics often necessitate the continuous use of vasopressor infusion [29]. Vasopressors can affect arterial tone and right ventricular function. Most patients (31/34) in this study were receiving norepinephrine infusion at the time of IVC clamping. We divided the patients into two groups according to whether the continuous norepinephrine infusion dose was less than 0.1 µg/kg/min or greater or equal to these values. When comparing variables, ΔRDII, SVV, and PPV changed significantly when norepinephrine was less than 0.1 µg/kg/min (or none), while these variables did not significantly change when norepinephrine infusion was ≥0.1 µg/kg/min group. Although the reason for this result is not clear, it may be attributed to the influence of catecholamine on vascular tone, which resulted in reduced PPV and SVV [30,31]. ΔRDII may not be directly affected by the infusion of catecholamine. Of interest, however, ΔRDII showed relatively poor performance in the group receiving greater than or equal to 0.1 µg/kg/min of norepinephrine (*p* = 0.093). We hypothesized that the reasons for this discrepancy were as are follow. The use of substantial doses of norepinephrine before vena-cava clamping, already risks hemodynamic instability and the necessity for a massive fluid infusion by the anesthesiologist. Thus, these factors might mask the real intravascular volume deficit. The variable from the Swan-Ganz catheter (CO_SG_ and SV_SG_) was significantly changed under norepinephrine greater than or equal to 0.1 µg/kg/min. Unfortunately, the use of the Swan-Ganz catheter is limited in general clinical situations due to its complexity [32].

Although ΔRDII monitoring appears quite attractive, our study has several limitations that must be acknowledged. First, current study was relatively small size, retrospective design, and not a real-time analysis. Caution should be granted given the non-Gaussianity of the distributions and the relatively small number of subjects. If automated-software could be developed for real-time measurement, it might prove a more useful noninvasive method to predict intravascular volume status and cardiac output change during general surgery [33]. A trial comparing automated method (using MATLAB software) versus manual determination of R-wave amplitude method has been conducted [28]. Secondly, technically, R-amplitude measurement using software is not always possible. If the raw ECG data demonstrated baseline wandering, some ECGs could not be appropriately measured even after a high-pass filter. Proposed methods from previous report which incorporate the removal of baseline wandering [34] or denoising could result in more accurate analyses. We recommend cautious interpretation should be always considered for the use of ΔRDII in the clinical application. Another important limitation of our ΔRDII analysis was that it is not suitable for open-heart surgery. Opening the thoracic cavity nearly always influences hemodynamic cardio-pulmonary interaction [35]. It is difficult to generalize our results to other clinical situations because hepatic failure patients present with unique cardiovascular physiology. Further study will be required to investigate ECG variation during general surgery, including during lateral or prone position.

In conclusion, ΔRDII is associated with changes in cardiac output and PPV in the preload-dependent state, while such a relation was not found in the preload-independent state. Although there remain limitations of these data regarding clinical applications, respiratory changes in ECG R-wave amplitude can be a surrogate indicator to assess intravascular volume status. We believe this simple and noninvasive monitoring method will contribute to fluid management in the operating room, with the caveat that cautious interpretation is necessary when high doses of vasopressors are used.

## Figures and Tables

**Table 1:** Demographic and clinical characteristics of the 34 patients studied.

Characteristics	
Patient characteristics and comorbidities	
Sex, Male (%)	23 (67.6)
Age (years)	52.7 ± 9.5
Weight (kg)	64.6 ± 10.7
Height (cm)	166.5 ± 7.8
BMI (kg/m^2^)	23.3 ± 3.4
Cardiovascular disease ^a^	4 (11.4)
Hypertension	5 (14.2)
Diabetes mellitus	12 (34.3)
Child-Pugh score	8.4 ± 2.3
MELD ^b^ score	15.5 ± 6.9
Disease	
Viral hepatitis-related ESLD ^c^	21 (61.7)
Alcoholic cirrhosis	6 (17.6)
Others	7 (20.6)
Drug	
Beta-blocker	11 (32.4)
Furosemide/spironolactone	21(61.8)/18 (52.9)
Echocardiographic findings	
Left ventricle mass index (g/m^2^)	89.4 ± 20.2
Left ventricular ejection fraction (%)	65.0 ± 5.5
Left ventricle internal dimension at end diastole (mm)	50.8 ± 8.2
Left ventricular posterior wall thickness at end diastole (mm)	8.7 ± 1.1
Interventricular septal dimension at end diastole (mm)	8.4 ± 1.8
E/E’ ratio ^d^	9.0 ± 2.4
Intraoperative norepinephrine use (%)	31 (91.2)

Data are expressed as the mean ± standard deviation or number (percentage). ^a^ Including prior myocardial infarction, angina pectoris, left ventricular dysfunction, and peripheral arterial occlusive disease. ^b^ MELD: Model for End-Stage Liver Disease ^c^ ESLD: end stage of liver disease. ^d^ Ratio of early transmitral flow velocity to early diastolic velocity of the mitral annulus. BMI, body mass index.

**Table 2:** Comparison of ΔRDII and other variables during IVC clamping (*n* = 34).

	Before	After	*p*-Value
MAP (mmHg)	88 ± 13	78 ± 9	<0.001
Heart rate (bpm)	84 ± 12	87 ± 14	0.007
CVP (mmHg)	11.6 ± 3.4	10.7 ± 3.7	0.197
PCWP (mmHg)	19.9 ± 5.0	16.3 ± 4.5	0.001
CO_SG_ (L/min)	7.0 ± 2.0	6.2 ± 2.0	<0.001
SV_SG_ (mL/beat)	86 ± 27	77 ± 28	<0.001
CO_FT_ (L/min)	6.4 ± 1.7	5.8 ± 1.7	0.001
SV_FT_ (mL/beat)	77 ± 24	69 ± 26	0.011
SVV (%)	6.1 ± 3.3	9.7 ± 4.9	<0.001
PPV (%)	7.0 ± 4.4	11.3 ± 5.6	<0.001
ΔRDII (%)	11.1 ± 6.1	14.2 ± 7.8	0.002
Frequency analysis Area under curve (mVolt•√Hz)			
Respiratory frequency	0.092 (0.018, 0.218)	0.139 (0.025, 0.314)	0.016
Cardiac frequency	1.670 (0.700, 2.750)	2.013 (1.059, 3.542)	0.001

Data are expressed as the mean ± standard deviation or median (interquartile range)., MAP, mean arterial pressure; CVP, central venous pressure; PCWP, pulmonary wedge pressure; COSG, stat mode of continuous cardiac output from Swan-Ganz catheter; SVSG, stroke volume from Swan-Ganz catheter; COFT, cardiac output from arterial pressure waveform analysis; SVFT, stroke volume from arterial pressure waveform analysis; SVV, stroke volume variation; PPV, pulse pressure variation; ∆RDII, respiratory variation of lead II R-wave amplitude.

**Table 3:** Comparisons of ΔRDII and other variables according to the dose of continuous norepinephrine infusion during inferior vena cava clamping.

	Norepinephrine < 0.1 μg/kg/min or None			Norepinephrine ≥ 0.1 μg/kg/min		
		*n* = 26			*n* = 8	
	Before	After	*p*-Value	Before	After	*p*-Value
CO_SG_ (L/min)	6.8 (5.8;7.3)	5.7 (4.8;6.7)	0.002	6.5 (5.8;8.5)	5.5 (5.8;8.5)	0.014
SV_SG_ (mL/beat)	84.0 (66.5;112.0)	76.0 (60.8;93.3)	0.003	78.5 (57.3;101.5)	57.5 (48.0;96.0)	0.042
CO_FT_ (L/min)	6.4 (4.6;8.3)	5.9 (4.0;7.4)	0.003	6.4 (5.5;7.6)	6.4 (5.1;6.9)	0.061
SV_FT_ (mL/beat)	76.5 (58.8;98.8)	66.5 (46.5;86.8)	0.013	65.5 (56.5;99.0)	64.0 (48.5;96.6)	0.400
CVP (mmHg)	12.0 (9.9;14.3)	10.7 (8.7;12.3)	0.153	10.8 (6.2;13.8)	10.4 (8.4;13.3)	0.917
PCWP (mmHg)	19.5 (15.8;22.6)	14.8 (12.6;18.7)	0.000	20.4 (15.8;26.2)	20.0 (14.8;25.0)	0.236
SVV (%)	4.7 (3.2;7.6)	8.9 (5.9;11.0)	0.000	6.9 (3.9;13.0)	13.3 (6.0;15.3)	0.107
PPV (%)	5.6 (3.5;8.8)	10.2 (7.4;14.9)	0.000	6.3(3.2;12.8)	11.8 (5.0;14.9)	0.234
ΔRDII (%)	10.9 (5.6;17.8)	13.5 (8.4;19.1)	0.032	12.3(5.5;16.7)	14.1(6.7;28.8)	0.093

Data are presented median (interquartile range). COSG, stat mode of continuous cardiac output from Swan-Ganz catheter; SVSG, stroke volume from Swan-Ganz catheter; COFT, cardiac output from arterial pressure waveform analysis; SVFT, stroke volume from arterial pressure waveform analysis; SVV, stroke volume variation; PPV, pulse pressure variation; ΔRDII: respiratory variation of lead II R-wave amplitude.
